# Successful internal fixation of a pelvic ring fracture during pregnancy: a multidisciplinary case report

**DOI:** 10.1186/s12884-025-08101-1

**Published:** 2025-10-06

**Authors:** Diana Schrick, Sándor Márton, Csaba Csontos, Diana Mühl

**Affiliations:** Department of Anaesthesiology and Intensive Care, Medical School, University of Pécs, Pécs, Hungary

## Abstract

**Background:**

Severe pelvic trauma during pregnancy is rare and requires concurrent maternal and fetal management. Multidisciplinary coordination is essential, yet reports of definitive pelvic fixation with the fetus in utero are exceptionally uncommon.

**Case presentation:**

A 28-week pregnant woman with no prior medical history sustained severe injuries (Injury Severity Score: 16) in a high-energy collision. Diagnostic imaging revealed a comminuted, displaced right hip fracture, sacral avulsion fractures, right-sided sacroiliac joint diastasis, and a large pre-sacral pelvic hematoma. Abdominal ultrasound confirmed a viable fetus with no signs of distress and no placental hematomas. The patient was admitted to intensive care unit for preoperative optimization, including fluid resuscitation, transfusion, and pain management. Continuous maternal monitoring and daily fetal assessments were performed. Following multidisciplinary consultations with obstetricians, neonatologists, traumatologists, and intensive care specialists, temporary right lower limb traction was applied, followed by closed reduction and screw osteosynthesis of the iliac bone and sacrum. Surgery was conducted with a perinatal team prepared for emergency cesarean delivery. No intraoperative fetal distress was observed. Postoperative recovery was uneventful, and the patient subsequently delivered a healthy male infant at term.

**Conclusion:**

This case contributes to the limited body of literature describing successful internal pelvic fixation during pregnancy with fetal retention until term. It illustrates that, with vigilant monitoring and collaborative, protocol-driven management, definitive orthopedic intervention can be planned and performed deliberately while achieving favorable maternal and fetal outcomes.

## Background

High-energy injuries (for instance, traffic accidents, most commonly) often combine with pelvic ring fractures (PRFs) during pregnancy as a ’locus minoris resistentiae’, which is a rare yet severe condition for both the mother and the fetus [1]. Prevalence of pelvic ring injury within blunt trauma-induced fractures is reported to be 5–8%^2^. Mortality rates are reported as 9% for the mothers and up to 60% for fetuses. There is a wide variety of these because several factors influence the mortality rate (severity of trauma, hemodynamic instability, pre-existing comorbidities, associated injuries, delay in diagnosis and treatment, gestation age at injury, placental injury, direct fetal trauma) [3–5]. Pelvic ring injuries result in hemorrhage, thus hypovolaemia that independently escalates patient mortality, irrespective of the underlying diseases. This risk is notably exacerbated in pregnant women because of flow-dependent uteroplacental circulation [6, 7]. Retrospective data indicate that surviving fetuses from mothers with fractures are more susceptible to premature birth, low birth weight, and enduring neurocognitive dysfunction [8, 9]. Previously published studies have revealed numerous uncertainties in the care of these patients [10]. Although maternal stabilisation is the primary focus in trauma management – reflecting the principle that maternal wellbeing underpins fetal well-bing – gestational age, anamnestic data from the pregnancy should be considered as well [1, 11].

## Case presentation

**Fig. 1:**
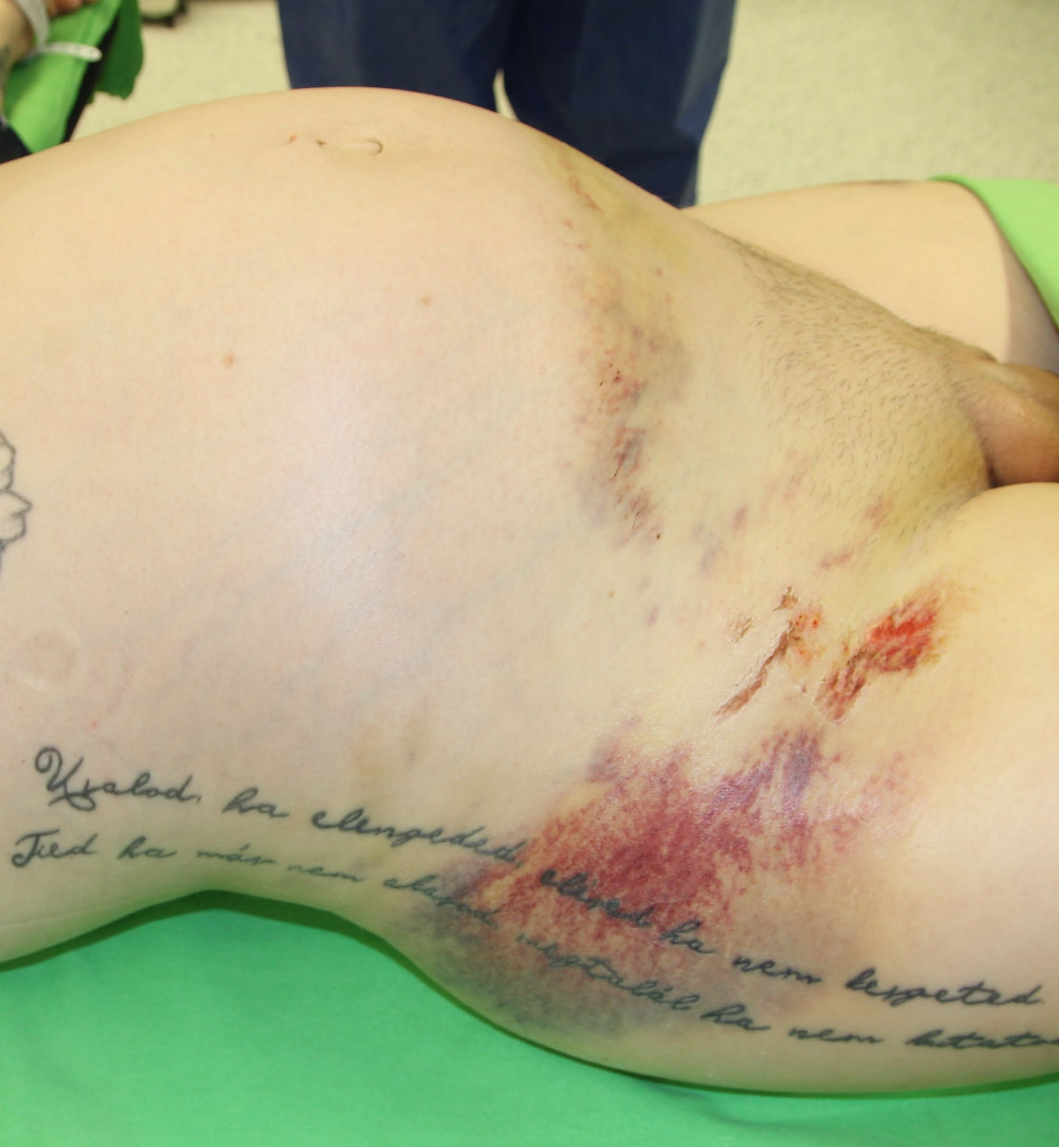
Indirect sign of pelvic injury after the removal of the pelvic belt

A 28-year old woman without any chronic disease suffered a traffic accident in company with her husband. The mechanism of injury involved a swerve during an overtaking maneuver, resulting in a collision with a tree. The injured woman was pregnant with a 27-week-old fetus. Seatbelts were fastened, but she did not use the belt deflector, and the airbags did not open. The first medical contact happened within 25 min at the accident site. Upon documentation, the oxyologist finds her with moderate hypotension (BP_sys_ 88Hgmm) and tachycardia (HR 110/min). After removing the patient from the wreck, she was admitted to the nearest county hospital emergency department on a long spine board, cervical collar, and pelvic belt. Upon arrival to the emergency department, during the initial assessment she was 15 on the Glasgow Coma Scale (GCS), and had a complaint of severe pain (8 on visual analogue scale; VAS). Upon primary examination, the patient was ABCDE unstable (tachycardia, deeper hypotension was detected), physical examination revealed indirect signs of pelvic injury (hematoma and pain) [12] as depicted in Fig. 1.

### Investigations

An emergency gynecological exam was performed [13] transabdominal ultrasound with no sign of retroplacental hemorrhages, and the cardiotocogram showed normal baseline tone of the uterus, profilactic intravenous magnesium was given for tocolytic effect.

**Fig. 2:**
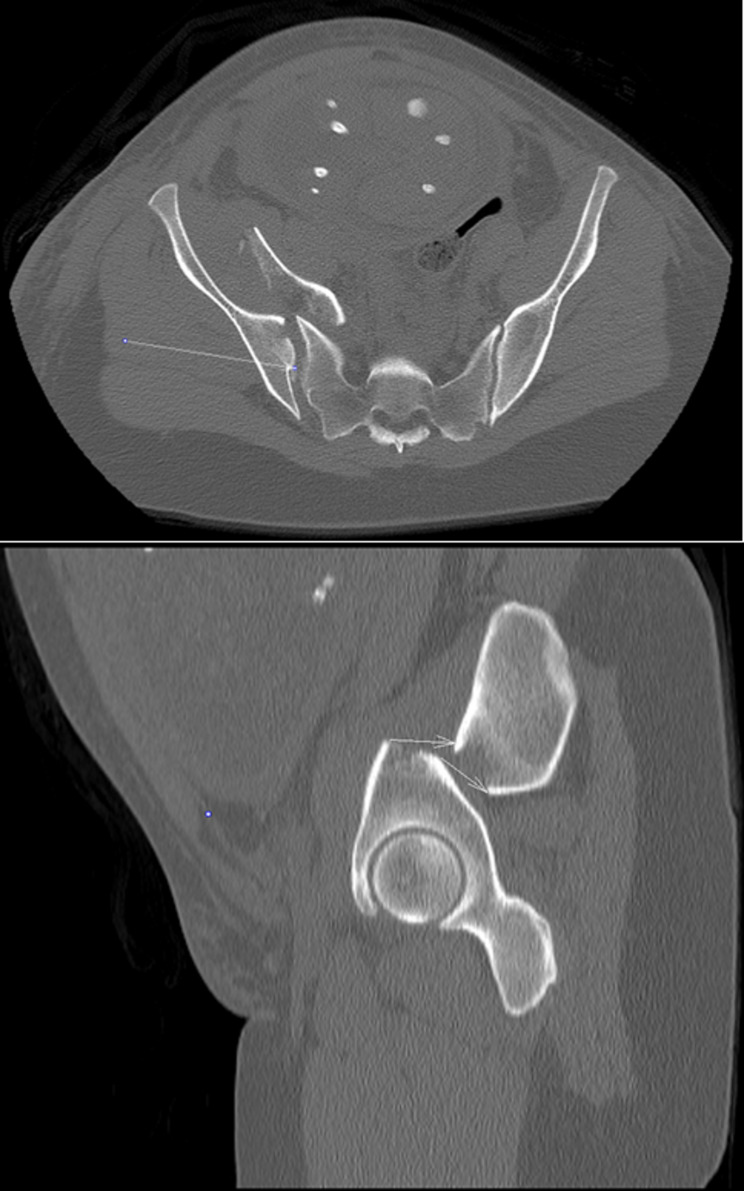
2D CT scan axial and sagittal views represent the fracture of the os ilium with complete bone width dyslocation and sacroiliacal joint dehiscence, anteriorly fetal parts can be seen

**Fig. 3:**
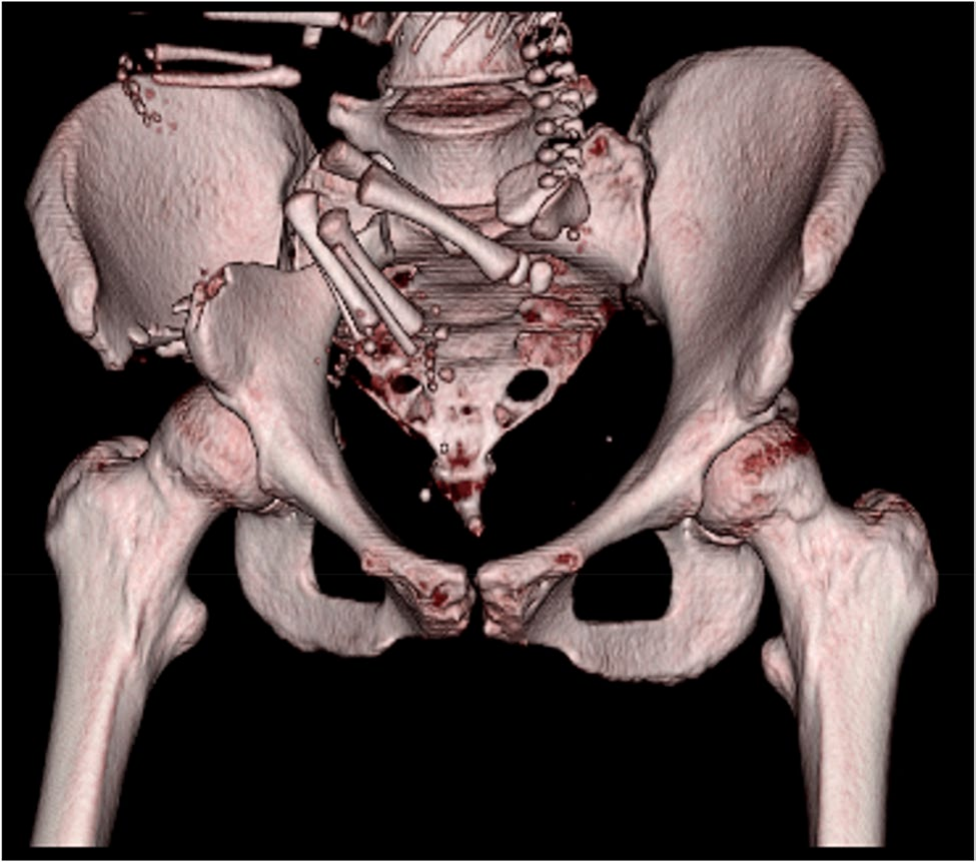
3D-image reconstruction of pelving ring fracture with the fetus in situ

During stabilization of the patients vital parameters, analgesia was provided with intravenous opioids Furthermore, an epidural catheter was inserted in the lateral decubitus position at the level of lumbal 3–4 vertebrae to maintain sufficient analgesia (with bupivacaine 0.25%) for the mother and sustain consistent uteroplacental blood flow to the fetus via vasodilatation. Fluid administration was performed with balanced crystalloids [14]. To prevent excessive hemodilution after bleeding, upon strict stratification of costs and benefits, blood transfusion (with 1 unit, ~ 200 ml type- and cross-matched red blood cell concentrate) was performed to maintain sufficient delivery of oxygen to end-organs while minimize the immunization risks. A low-dose CT scan was performed to detect the exact nature of the injuries. Imaging revealed a fracture of the right iliac bone with full dislocation and complete displacement of the sacroiliac joint, and a fracture of the massa lateralis of the sacrum with significant periosteal hemorrhage, causing a slight dislocation of the uterus, as represented in Fig. 2. in axial and sagittal views. During transfer to the higher-level care centre (Department of Anaesthesiology and Intensive Care, University of Pécs Medical School, Clinical Centre), a clinical radiologist performed 3D image reconstructions to support planning further treatment. As shown in Fig. 3. these images revealed a complex pelvic ring fracture with part of the fetus visible within the maternal pelvis.

### Treatment

The patient was admitted to the Central Intensive Care Unit of the University of Pécs Medical School for preoperative optimization and perioperative care.

During observation, daily obstetric assessments, including ultrasound (and flowmetry) was performed regularly. In consultation with the obstetric team, antenatal corticosteroids - specifically dexamethasone 24 mg intramuscularly, administered as four divided doses of 8–4–8–4 mg at 12-hour intervals - were given to promote fetal lung maturation in anticipation of a possible emergency cesarean section. In addition, anti-D immunoglobulin prophylaxis (300 µg intramuscularly) was administered, as feto-maternal hemorrhage could not be definitively excluded following the high-energy injury, and the prophylaxis was therefore warranted to prevent Rh sensitization.

Clinical pharmacologists and clinical microbiologists were involved in the daily decision-making in the patient’s pharmacotherapy. The patient received prophylactic amoxicillin–clavulanic acid at a dose of 1200 mg, twice daily. To minimize the usage of opioids during pregnancy, an epidural catheter was used, and local anesthetic (bupivacaine 0.25–0.5%) was administered to the patient via a patient-controlled analgesia (PCA) pump. Furthermore, paracetamol (1000 mg) was used regularly (maximum 3000 mg within 24 h). For procedures associated with increased pain, such as mobilization and specific nursing interventions, the patient was administered on-demand bolus doses of either fentanyl (50 µg) or sufentanil (2–5 µg), given separately at different times according to clinical need.

Venous-thromboembolic prophylaxis was executed with enoxaparine 4000 IU once a day, respectively. A H2 receptor blocker (famotidine 40 mg, twice daily) was used for the ulcer prophylaxis.

Further blood transfusions were performed (with 2 unit type- and cross-matched red blood cell concentrate) to correct maternal anemia and optimize fetal oxygen delivery. Prothrombin-complex concentrate (PCC 1000 IU) and fibrinogen (2 g) were administered before the operative management upon thromboelastic examination to prevent bleeding.

During the preparation for the operation, the traumatologist inserted an extension into the condyles of the right femur and performed traction with 5 kg to release the acetabulum from pressure.

In our clinical center, there is a block system, which means means that specialty hospitals are located in separate buildings within the city. This posed a challenge in determining the optimal surgical location - either the Department of Obstetrics, which would be functionally advantageous in the event of obstetric complications, or the Department of Traumatology, which is co-located with the intensive care unit and thus offers technical advantages. Following multidisciplinary consultation, the surgery was scheduled at the traumatology operating centre, with an obstetrician, a neonatologist, and a neonatal emergency team present to address any potential complications [15].

We administered local anesthetic (100 mg lidocain) into the epidural space to perform opioid-sparing anesthesia. A lumbar epidural produces symphathetic blockade below the level of the block, causing arterial and venous vasodilation in the lower body. That can lower systemic vascular resistance and mean arterial pressure; if hypotension occurs, uterine blood flow falls because the uteroplacental circulation is not autoregulated, it is largely pressure-dependent. Though, an effective neuraxial analgesia reduces maternal catecholamines – reduced pain and stress are associated with lower circulating levels of epinephrine and norepinephrine - which can improve intervillous-uteroplacental flow [16, 17].

**Fig. 4:**
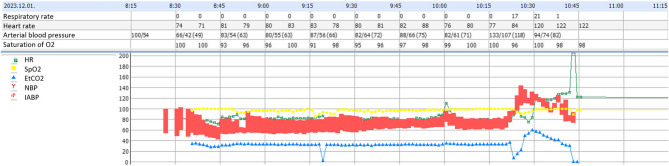
Vital parameters of the patient during anesthesia; abbreviations – HR: heart rate, SpO2: peripheral oxygen saturation, etCO2: end-tidal carbon dioxide, NBP: non-invasive blood pressure, IABP: intra-arterial blood pressure

Balanced, fast-track anesthesia with a low minimum alveolar concentration (MAC) of anesthetic gases was administered, allowing extubation in the operating theatre immediately after surgery. This modern practice (fluids, left uterine dysplacement and use of vasopressors – often phenylefrin) aim to keep MAP (mean arterial pressure) near baseline, which preserves uteroplacental perfusion and associated with good fetal acid-base status [18]. The patient was hemodynamically stable during the whole procedure, and there was no sign of fetal compromise. Intraoperative vital parameters are shown in Fig. 4.

**Fig. 5:**
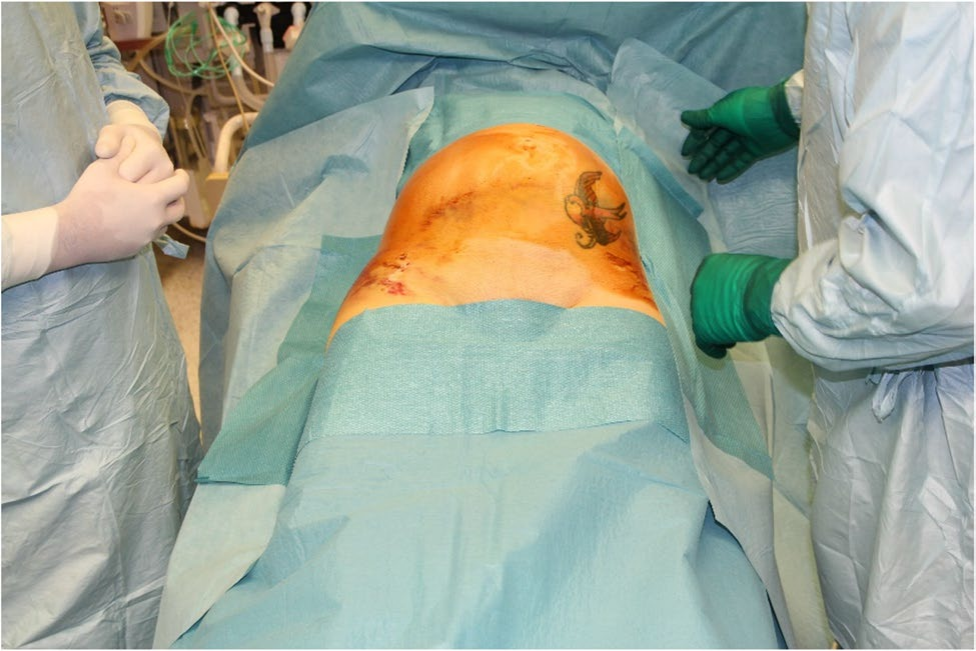
Left lateral tilt position at the beginning of the surgery

**Fig. 6:**
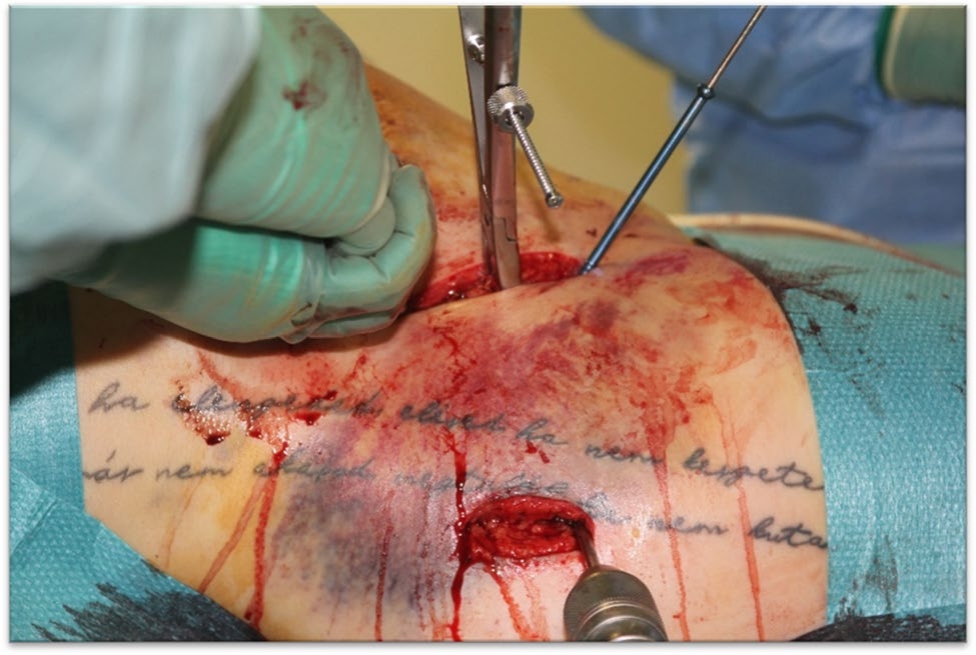
Minimal invasive operative pelvic reconstruction (intraoperative photo documentation)

**Fig. 7:**
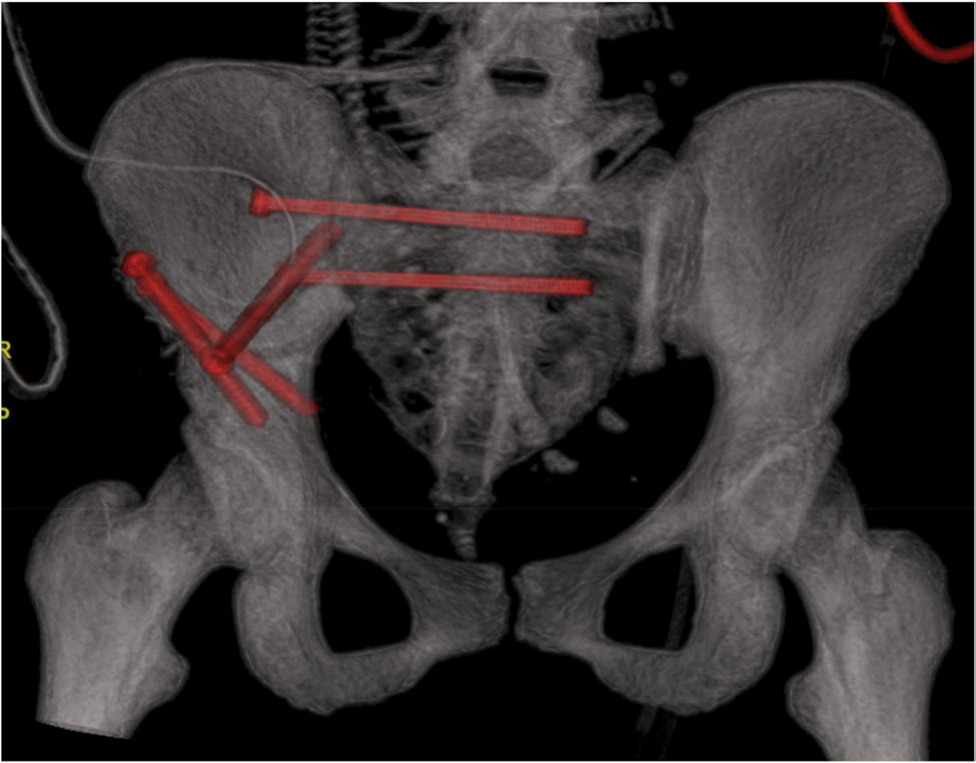
CT 3D reconstruction image of the postoperative pelvic state, 5 osteosynthetic screws in situ (labelled with red)

Operative treatment held some challenges as well, because the operation site was extremely close to the womb, with limited maneuvering capacities. Achieving the optimal patient position proved challenging. Finally, the patient was tilted left laterally to prevent the compression of the inferior vena cava (resulting in low preload and cardiac output syndrome) and to let the surgeons operate more freely, as shown in Fig. 5. The operating site was identified under an image-intensifier (X-ray). The usage of ionizing radiation was minimized during the procedure; overall radiation time was 3 min and 3 s, with 9.47 Gy cm2. Traumatologists performed the repositioning and reconstruction of the fracture of the os ilium with full bone width disclocation after a longitudinal incision, presented in Fig. 6. Above the tip of the right greater trochanter, then penetrating the fracture gap with a Steinmann nail, drilling into the distal fragment of the second nail, and moving upward, pulling it up like a joystick next to the uterus. Lysis of the sacroiliac joint was reconstructed with screw osteosynthesis, by drilling two transverse screws into the massa lateralis part of the sacrum. The postoperative control CT scan, shown in Fig. 7, depicts the position of the inserted screws (highlighted in red) and demonstrates the restored postoperative anatomy of the female pelvis.

Postoperative observation was without any complications. Inflammation parameters showed a kinetics to systemic inflammatory response, so the empiric antimicrobial therapy was continued. The postoperative fetal diagnostics (cardiotocograph, ultrasound) verified fetal well-being.

As a residual complication of the injury, the patient experienced numbness on the lateral part of the right lower limb, the neurologist council suggested compression of the sciatic nerve due to the pelvic hematoma. Unfortunately, the MRI could not be performed because the patient had non-MRI-compatible metals in her ankle (previous malleolar osteosynthesis). Physiotherapy and a transcutaneous nerve stimulator were introduced as soon as possible with adequate analgesia. Based on the findings, the patient did not need further multiparametric observation and was discharged to the territorially competent hospital, equipped with traumatology and obstetric facilities, for further observation and early rehabilitation.

### Outcome and short term follow-up

After early mobilization, the patient was discharged home. She was able to move only with walking aids until the term of pregnancy. At term (on the 39th gestational week) her pregnacy was resolved by elective cesarean section - due to the previous pelvic operation and the breech position of the fetus – a healthy male neonate was delivered, with the Apgar Score of 8/10. Her late comprehensive musculoskeletal rehabilitation occured after the postpartum period, together with her baby, the rehabilitation was successful the young lady is able to live her life independently with full accessibility. The regular neurodevelopmental assessment revealed findings within normal limits for his age.

## Discussion

Pelvic ring injuries in pregnant women present significant risks to maternal and fetal health.

Early recognition, stabilization, and vigilant monitoring of maternal vital signs and fetal wellbeing are crucial. Vaginal bleeding/discharge serves as a critical fetal risk indicator in most of the patients [19]. Effective management of such complex cases relies on multidisciplinary collaboration and carefully tailored interventions.

**Fig. 8:**
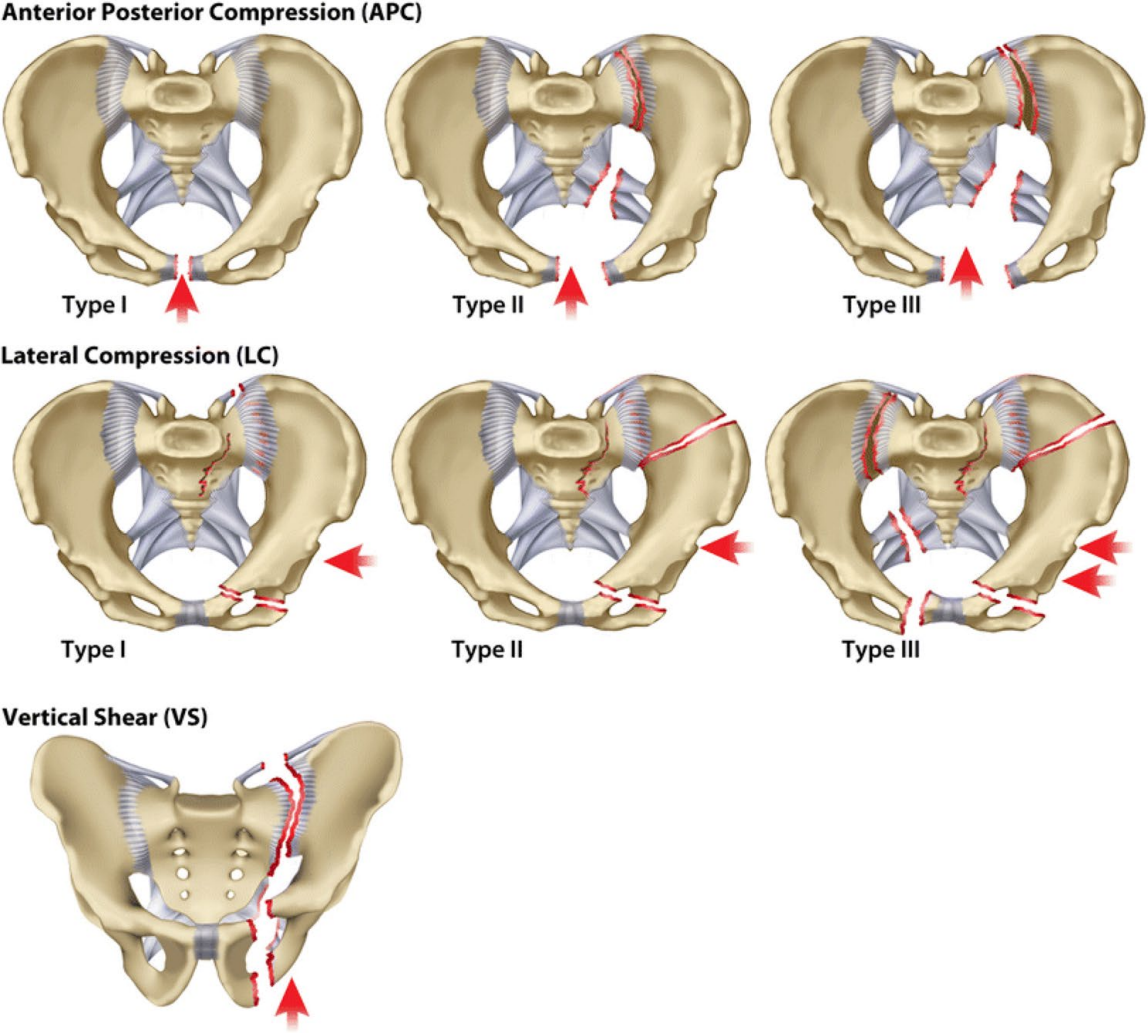
Young and Burgess Young and Borgess classification of Pelvic Ring Injuries. Photo originates from Alton et al. A.O. Classifications in Brief, published in Clin Orthop Relat Res, 2014^23^

Various classifications have been proposed for pelvic ring fractures. Combining the commonly used osteoligamentous pelvic ring injury classification schemes (AO/OTA and Young-Burgess) seems useful in acquiring information on mechanical and haemodynamic instability. The widely accepted Arbeitsgemeinschaft für Osteosynthesefragen classification system (with the 2018 revision) is used to determine the stability of the pelvic ring: type A with a stable pelvic ring, type B with a partially unstable pelvic ring, and type C with a volatile pelvic ring [20]. While the Young and Burgess classification is a modification of the earlier Tile classification, which takes into account force type, severity, direction, as well as injury instability [21–23] shown in Fig. 8.

Management of a pregnant patient is always a challenge, to save two lives in parallel (mother & fetus), and may pose a dilemma for a medical practitioner to choose between them (the mother’s lives have top priority) [24]. Our case report aims to draw attention to the importance of multi-disciplinary management, care of patients, and the usage of different international guidelines to individualize treatment strategies to effectively manage the complexities associated with pelvic injuries during pregnancies to provide a better outcome.

Pelvic fractures in pregnancy are rare, resulting in a paucity of evidence-based management.

Controlled hypotension is recommended transiently to reduce the risk of bleeding, but maintaining normal uteroplacental blood flow is vital. The limited usage of vasopressors (first-choice noradrenaline) has been reported to reduce uterine blood flow. However, noradrenaline seems to have no detrimental effect on the well-being of the fetus [25]. With fluid administration (preferably balanced crystalloids), we must be careful to prevent hemodilution. A liberal approach to blood transfusion methods is essential to provide enough oxygen delivery with the least risk of immunization of the mother. To prevent trauma-induced coagulopathy, bedside point-of-care hemostasis assays should be performed regularly, and interventions should be performed at the exact points of hemostasis if needed, using exact substrates (fibrinogen, PCC). To reduce oxygen consumption, adequate analgesia with a multimodal approach is essential.

Non-obstetric surgery may be necessary during any trimester of pregnancy, presenting the unique challenge of managing the health of both the mother and the fetus. The second trimester is generally regarded as the period of lowest risk for preterm birth, and by the eighth week of gestation, the critical stages of embryonic development are largely complete In the third trimester, administration of antenatal corticosteroids - such as dexamethasone or betamethason - should be undertaken in consultation with the relevant specialists to enhance fetal lung maturation [26, 27]. Non-steroidal anti-inflammatory drugs should not be used during pregnancy, unless the expected benefits to the mother outweigh the risk to the fetus and must limit the use to the lowest effective dose to the shortest duration, though these drugs are contraindicated in the third trimester of pregnancy to prevent the closure of premature patent ductus arteriosus and the formation of oligohydramnion and potential postnatal kidney dysfunction [28–30].

The American College of Obstetricians and Gynecologists (ACOG) recommends fetal heart rate (FHR) measurement using intermittent or continuous cardiotocography (CTG) before and after any given surgery, regardless of gestational age of the fetus, furthermore fetal heart rate (FHR) can be measured by sonography as well [31, 32]. Most anesthetic agents cross the placenta and may affect fetal heart rate tracing, causing reduced variability. Fetal monitoring helps optimise maternal positioning (left lateral tilt to prevent vena cava compression syndrome; first described by Crawdord et al. in 1972 and still supported by the ERC as well is 2021) oxygenation, ventilation, and hemodynamic management of the mother [11, 33].

Thus pelvic trauma during pregnancy is rare, the long-term consequences on successive pragnancy is still unknown. Neither the American College of Obstetricians and Gynecologists nor the European Board and College of Obstetrics and Gynaecology have established clear recommendations regarding the mode of delivery following a pelvic fracture. However, a few case reports and review articles have addressed this challenging question, emphasizing the need for individualized, multidisciplinary decision-making based on maternal mobility, the time elapsed since injury, and the type of surgical repair. In 2015, *Stohlner et al.* published a case report on external fixation for pelvic ring injury during pregnancy [34]. The article demonstrated a successful use of external fixation for initial temporary stabilisation and subsequent definitive management of a pelvic ring fracture in late pregnancy, which allowed the fetus to remain in utero until mature enough for delivery. *Mennen et al.* recently published a retrospective observational study [35] revealed that women with retained hardware after pelvic ring fixation could have vaginal delivery with a rare occurance postpartum complications, 168 fertile age patient were enrolled into this cited research of whom 13 had a pregnancy after surgical stabilization of pelvic ring fracture. Seven women had a total of 11 vaginal deliveries, and six women had cesarean section. The decision for vaginal delivery was often the wish of the mother (*n* = 4; 57%) while the decision to opt for cesarean section was made by the surgeon or obstetrician (*n* = 5; 83%). One woman in the vaginal delivery group suffered a postpartum complication possibly related to her retained pelvic hardware. Thus, the rate of primary cesarean sections is still high (46%), which might be influenced by physician bias. *Lewis et al.* summarized the current literature and there was a perceptible increase in the percentage of women with CS after pelvic trauma, at this time there is no clear evidence to support elective cesarean section as the sole indication after a prior pelvic fracture, but it should be taken into considarion the multidisciplinary opinions (orthopedist and obstetrician) alongside all together with the best available evidene available [36, 37].

Several issues need to be addressed when establishing perioperative management of a severely injured pregnant patient; these are listed in Table 1.

**Table 1:** Bullet points for the management of pelvic ring injuries in late pregnancies

1. Multimodal monitoring of the mother and the fetus
2. Adequate analgesia to reduce the oxygen consumption
3. Optimize fluid status and hemodynamics to maintain normal oxygen delivery to tissues
4. Assess timing and mode of delivery
5. Assess timing and mode for reconstructive surgery
6. Choose an ideal anesthetic technique: neuraxial, regional anesthesia if possible
7. Continue postoperative monitoring of the mother and the fetus
8. Start mobilization as soon as possible, and prevent thromboembolic events
9. Regular postnatal medical check-ups to detect any neurodevelopmental problems, and to initiate early intervention if needed

This case report contributes to the limited body of literature describing successful internal fixation during pregnancy with fetal retention until term [35, 38–40]. Our patient had a cesarean section on the 39th gestational week of her pregnancy, and she gave birth safely to a healthy male infant (with Apgar 8/10). During the regular check-ups, doctors and nurses inspect the child’s status (it was performed in the early neonatal age, and then in the 1 st, 2nd, 3rd, 4th, 6th, 12th, and 15th months after birth, according to the governmental regulation). The infant’s physical and neurocognitive development was consistent with age-appropriate milestones, with no delays identified relative to peers; therefore, further neurodevelopmental assessment was deemed unnecessary.

### Limitations of this case report

This case report has several limitations. As a single-patient observation, the findings cannot be generalized to all pregnant trauma patients with pelvic fractures. Intraoperative and postoperative assessments did not include direct quantitative measurements of uteroplacental perfusion, limiting the ability to objectively confirm the theoretical benefits of certain anesthetic interventions. Long-term follow-up of the child was restricted to routine developmental milestone assessments, without standardized neurocognitive testing. Finally, the management approach was influenced by the specific resources and infrastructure of our institution, which may differ from those available in other settings.

## Data Availability

All relevant patient data are included anonymously in the manuscript. Additional anonymized clinical records (available only in Hungarian) may be provided by the corresponding author upon reasonable request.

## Abbreviations

2D: 2-dimension
3D: 3-dimension
ABCDE: Airway-breathing-circulation-disability-exposure
ACOG: American College of Obstetricians and Gynecologists
AO: Arbeitsgemeinschaft für Osteosynthesefragen
APC: Anterior posterior compresion
CS: Cesarean section
CT: Computed tomography
CTG: Cardiotocography
EBM: Evidence-based medicine
etCO2: End-tidal carbon dioxide
FHR: Fetal heart rate
GCS: Glasgow Coma Scale
HR: Heart rate
IABP: Intra-arterial blood pressure
ISS: Injury severity score
LC: Lateral compression
MAC: Minimum alveolar concentration
MRI: Magnetic resonance imaging
NBP: Non-invasive blood pressure
OTA: Orthopaedic Trauma Association
PCA: Patient controlled analgesia
PCC: Prothrombin-complex concentrate
PRF: PELVIC ring fractures
SpO2: Peripheral oxygen saturation
VAS: Visual Analogue Scale
VS: Vertical shear
